# Current Strategies to Generate Human Mesenchymal Stem Cells In Vitro

**DOI:** 10.1155/2018/6726185

**Published:** 2018-08-26

**Authors:** Jennifer Steens, Diana Klein

**Affiliations:** Institute for Cell Biology (Cancer Research), University Hospital Essen, University of Duisburg-Essen, Essen, Germany

## Abstract

Mesenchymal stem cells (MSCs) are heterogeneous multipotent stem cells that are involved in the development of mesenchyme-derived evolving structures and organs during ontogeny. In the adult organism, reservoirs of MSCs can be found in almost all tissues where MSCs contribute to the maintenance of organ integrity. The use of these different MSCs for cell-based therapies has been extensively studied over the past years, which highlights the use of MSCs as a promising option for the treatment of various diseases including autoimmune and cardiovascular disorders. However, the proportion of MSCs contained in primary isolates of adult tissue biopsies is rather low and, thus, vigorous ex vivo expansion is needed especially for therapies that may require extensive and repetitive cell substitution. Therefore, more easily and accessible sources of MSCs are needed. This review summarizes the current knowledge of the different strategies to generate human MSCs *in vitro* as an alternative method for their applications in regenerative therapy.

## 1. Introduction

Among the adult stem cells, MSCs are supposed to be the most promising stem cell type for cell-based therapies [1–4]. Compared with less differentiated pluripotent stem cells, in particular embryonic stem cells or induced pluripotent stem cells (iPSCs), MSCs are well tolerated and lack ethical concerns as well as teratoma-formation and histocompatibility issues [5–7] [8, 9]. Adult MSCs are multipotent cells, which are commonly characterized by their ability to adhere on plastic, by the expression of a typical panel of MSC surface markers (CD105(+), CD73(+), CD90(+), CD11b(−), CD79a(−), CD19(−), and human leukocyte antigen (HLA-DR) (−)), and the ability to differentiate into mesenchymal and nonmesenchymal tissues in vitro and in vivo [10, 11].

Once therapeutically applied, MSC can either act directly by homing to particular anatomical sites after transplantation and differentiating into specific cell types to locally restore the damaged tissue. Even more important, MSCs can support tissue regeneration by a paracrine (“hit and run”) mechanism of action, such as secretion of multiple bioactive molecules capable of stimulating recovery of injured cells and inhibiting inflammation [12–14]. In addition, MSCs lack immunogenicity and possess the ability to perform immunomodulatory functions [15, 16]. These unique properties have promoted numerous applications of MSCs which currently undergo hundreds of clinical trials (http://www.clinicaltrials.gov) for disease treatments including graft versus host disease, chronic obstructive pulmonary disease, Crohn's disease, or even multiple sclerosis [17–20]. Genetically modified MSCs were further used to enable targeted delivery of a variety of therapeutic agents in malignant diseases [21–23].

The classical known reservoir of MSCs is the bone marrow, but nowadays, MSCs are effectively isolated from almost every organ such as adipose tissue, cartilage, muscle, liver, blood, and blood vessels [4, 24–29]. However, there are several limitations for the vigorous *in vitro* expansion of ex vivo isolated adult MSCs: a decline of their plasticity and *in vivo* potency over time was reported, as well as accumulated DNA abnormalities and replicative senescence [30–35]. In addition, variations of the quality of obtained donor cells and tissue sources have caused numerous inconsistencies in the reported *in vivo* effectiveness of MSCs [36–39]. Therefore, more reliable sources of MSCs remain an important problem.

To circumvent many of these issues, alternative methods to generate therapeutically sufficient numbers of MSCs *in vitro* were established. MSCs for autologous cell replacement therapy can be derived from immune-compatible somatic cells, which possesses huge clinical potential. However, the large-scale production of human MSCs for regenerative cell therapies depends on well-defined, highly reproducible culture and differentiation conditions. This review will focus on the different methods to generate therapeutically active MSCs *in vitr*o.

## 2. Patient-Specific MSCs

**Figure 1:**
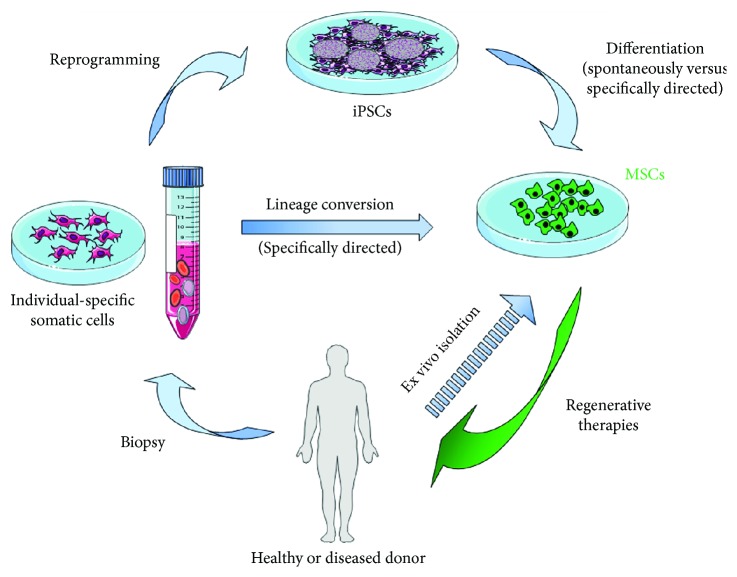
Patient-specific adult MSCs. Somatic cells (e.g., fibroblasts and peripheral blood cells) can be isolated from individual healthy or diseased donors (biopsy) and (i) directly converted into MSCs or (ii) reprogrammed into iPSCs by the introduction of the common transcription (Yamanaka) factors *OCT4*, *SOX2*, *KLF4*, and *c-MYC*. iPSCs were characterized by indefinite self-renewal and pluripotent differentiation capacities and, thus, represent an attractive source to generate unlimited cell numbers for targeted differentiation into MSCs. For regenerative therapy, only donor cells that have reached a particular differentiation stage could be used, which means that the iPSCs must first be brought to an ordered differentiation path. MSC differentiation of iPSC is initiated either spontaneously (by deprivation of pluripotent signals) or specifically directed by the induction of mesodermal differentiation, followed by treatment with MSC-specific growth factors that allows then the isolation and expansion of the selected MSCs under chemically defined cell culture conditions. As an alternative pathway, patient-specific somatic cells can directly programmed/transdifferentiated to MSCs which would avoid the need for prior reprogramming those cells back the pluripotent stage. Hypothetically, human MSCs could also be obtained by a direct programming approach, by ectopic expression of MSC-specific transcription factors in iPSCs and somatic cells, or by the introduction of cell type-specific microRNA molecules that functions in RNA silencing and posttranscriptional regulation of MSC gene expression. Morphology-based manual selection and/or sorting for cell type-specific cell surface markers using flow cytometry or immunomagnetic separation might further be used to increase purity of generated MSCs. The generation of patient- and disease-specific iPSCs is a valuable tool for regenerative therapies, for example, restoration of function through transplantation of ex vivo manufactured cells.

MSCs can be derived from different donor cells via 2 primary strategies: (1) direct conversion or induced transdifferentiation of patient-specific somatic cells or (2) differentiation from reprogrammed (pluripotent) somatic cells (iPSCs) (Figure 1). No matter which way of in vitro generation is chosen, MSCs emerge then from the proliferating donor cells in the presence of mesodermal growth factors, growth factor inhibitors, and small molecules. When iPSCs were used, MSCs can even be derived spontaneously by depriving the pluripotent signals from the culture conditions. In an additional, but up to now theoretical approach, derivation of MSCs could also be obtained by direct programming that would mean the ectopic expression of MSC-specific (transcription) factors that regulate MSC gene expressions [40–42]. To enrich the generated MSCs further, some forms of cell sorting and isolation using morphological features and/or antibodies specific for MSC-typical cell surface molecules or genetic tagging of the iPSCs with lineage-specific fluorescent reporter systems are required.

## 3. Human Embryonic Stem Cell-Derived MSCs

The first reports on MSCs generated from pluripotent stem cells were performed with pluripotent embryonic stem cells before iPSCs came into focus. Xu et al. isolated human embryonic fibroblast-like cells (HEF1 cells) from pluripotent human embryonic H1 stem cells after induction of differentiation by small aggregate formation (embryonic bodies) and subsequent cultivation in HEF1 medium (knockout- (KO-) DMEM supplemented with 10% heat-inactivated fetal bovine serum (FBS) and nonessential amino acids) [43]. The remaining fibroblast-like cells were further infected with a retrovirus expressing human telomerase reverse transcriptase (hTERT) which extended their replicative capacity, resulting in immortal human HEF1-hTERT cells. These cells exhibited a similar marker profile like MSCs and had the capacity to differentiate into cells of the osteogenic lineage, as telomerase expression in human MSCs had already been shown to enhance an osteogenic differentiation potential [44].

Only one year later, a more directed approach for the in vitro generation of MSCs was reported. Barberi et al. used the same embryonic H1 stem cell line (together with H9 embryonic stem cells) and cocultured these cells with the mesoderm embryonic cell line OP9 (mouse bone marrow stromal cells) to induce mesodermal differentiation in the presence of 20% heat-inactivated FBS (in alpha-MEM) for approximately 40 days prior flow-activated cell sorting (FACS) for the MSC marker CD73 [45]. This simple and quite unspecific differentiation protocol yielded multipotent mesenchymal precursors from human embryonic stem cells with typical average of 5% CD73-positive cells (in the mixed culture of OP9 and differentiated embryonic stem cells), which expressed several classical MSC markers and differentiation capabilities [45]. Gene expression profiling in addition confirmed a MSC-typical expression profile of differentiated MSC as compared to primary human bone marrow-derived MSCs including the MSC protein DSC54, neuropilin, hepatocyte growth factor, forkhead box D1, and notch homolog [45]. Thus, the basis for the *in vitro* generation of MSC differentiated from pluripotent stem cells which followed the classical MSC characteristics was made.

A number of reports followed to derive MSCs from human embryonic stem cells. A more specific approach was provided by Lian et al. who established a protocol for the derivation of clinically compliant MSCs, which were derived from Hues9 and H1 human embryonic stem cells without the use of animal products [46]. Mesodermal differentiation was induced by plating trypsinized embryonic stem cells in MSC growth medium supplemented with serum replacement medium, basic fibroblast growth factor (bFGF/FGF2), and platelet-derived growth factor AB (PDGF-AB) on gelatinized tissue culture plates. After one week of culture, CD105(+)- and CD24(−)-differentiated cells that comprised approximately 5% of the culture were sorted via FACS. Classical MSC characteristics were proven including gene expression analysis as compared to bone marrow MSCs [46]. In addition, the CD24-negative isolation allowed for the selection of the desired cells deprived from remaining non- or partially differentiated embryonic stem cells, as CD24 was identified as a human embryonic stem cell marker. Although the authors successfully reduced the unacceptable risks of tumorigenicity or potential xenozootic infection by circumventing the coculture with murine cells, the authors did not completely circumvent the use of animal products, namely, gelantin for coating and antibodies for flow cytometry purification.

This issue was addressed in the following study. Karlsson et al. established an optimized protocol resulting in the simple and reproducible derivation of mesenchymal progenitors from xeno-free, undifferentiated human embryonic stem cell lines [47, 48]. Therefore, undifferentiated embryonic stem cells were removed from the supporting feeder layer, enzymatically dissociated, and plated as high-density cultures on plastic dishes coated with human recombinant gelatin in medium supplemented with human serum (10%) and human recombinant bFGF. After 7 days, the resulting outgrowth of heterogeneous cell types was further passaged and cultured until a homogeneous culture with mesenchymal progenitor morphology (at passage 2-3) was achieved [48, 49]. Resulting MSC characteristics as well as microarray analysis confirmed the MSC nature of generated MSCs as compared to MSCs isolated from human bone marrow aspirates from the iliac crest [49]. Preclinical evaluation of implanted human embryonic stem cell-derived mesenchymal progenitor cells further revealed that generated cells gave rise to homogeneous, well-differentiated tissues that were exclusively of mesenchymal origin while no teratoma formation was observed [48]. The authors successfully established here a robust protocol that does not require cell transfection, coculture, cell sorting, or subjective manual selection for the xeno-free derivation of mesenchymal progenitors from diverse human embryonic stem cell lines that were safe for the use in tissue engineering and cell therapies.

Conclusively, MSCs can either be derived from human embryonic stem cells spontaneously, upon less stringent culture conditions, and in particular upon culturing in medium which is deprived of pluripotent signals, or by a specific stimulus (e.g., growth factors or inhibitors) which directs MSC differentiation. Most of these protocols were consistent and cost-effective, but inefficient, as the MSC population yielded by the unspecific differentiation methods yielded only approximately 5% MSCs. Pure cultures could then be established upon prolonged culturing, by fluorescence-activated cell sorting or by manual selection of cell populations.

## 4. iPSC-Derived MSCs

Human iPSCs constitute a well-characterized, generally unlimited cell source for the mass generation of lineage- and patient-specific MSCs without any ethical concerns because of their theoretical unlimited growth and differentiation potential. Human iPSC-derived MSCs were already shown to display similar features with mature MSCs at the genetic and functional levels [37, 50–52]. As already stated, the major challenge is here to establish reliable, efficient, and scalable protocols to differentiate functionally mature adult MSCs.

The classical method for differentiating iPSCs towards MSCs is the use of media that contains a high serum concentration or MSC-typical growth factors such as bFGF after mesoderm induction [32, 50, 53–55]. Induction of mesodermal differentiation is usually achieved by embryoid body formation or mesodermal-inductive factor treatments (*bone morphogenetic protein 4*, activin A/nodal, bFGF, and g*lycogen synthase kinase 3* inhibitors or WNT ligands) in chemically defined monolayer systems. Successive treatment with MSC-specific growth factors and/or sorting for MSC-specific cell surface markers using flow cytometry or immunomagnetic separation allows then the isolation and expansion of the selected MSCs under chemically defined cell culture conditions.

A clinically compliant protocol for the MSC differentiation of human iPSCs was established by Lian et al. [56]. According to their previously established protocol for the derivation of MSCs from human embryonic stem cells, the authors used three iPSC lines (iPSC(iMR90)-5 and iPSC(iMR90)-4 cells derived from IMR90 fibroblasts as well as iPSC(foreskin)Clone1 cells derived from foreskin fibroblasts), which were cultured on gelatin-coated plastic dishes in KO-DMEM supplemented with 10% serum replacement medium in the presence of bFGF, PDGF-AB, and epidermal growth factor to foster the MSC outgrowth [46, 56]. After 1 week of culturing, CD24(−) and CD105(+) cells were isolated via FACS and clonally expanded. By this, the authors successfully generated therapeutically active MSCs which exhibited the classical MSC characteristics and furthermore the ability for self-renewal in culture for >120 population doublings without obvious loss of plasticity or onset of replicative senescence [56]. The transplanted iPSC-derived MSCs were shown to be superior in attenuation of severe hindlimb ischemia (significantly improved vascular and muscle regeneration) than adult bone marrow MSCs which may result from a better in vivo survival and to their trophic factors that protect endangered cells after ischemic injury [56]. Giuliani et al. confirmed the beneficial role of iPSC-derived MSCs as compared to bone marrow MSCs concerning survival and longevity [57]. The authors used MSC differentiated from 5 iPSC lines (H9-iPS, SA-01-iPS, PB03, PB10, and PB11) using DMEM/F12 medium supplemented with 10% heat-inactivated FBS, bFGF, and nonessential amino acids as differentiation medium. Respective iPSC-derived MSCs displayed remarkable inhibition of natural killer (NK) cell proliferation and cytolytic function, while being more resistant than adult bone marrow MSCs to preactivated NK cells, which highlights their potential to prevent allograft rejection [57]. In line with these findings, a differential expression of ion channels in iPSC-derived MSCs was shown to contribute to their higher proliferation capacity compared with classically bone marrow MSCs [58]. Among the different ion channels, increased expression of the functional *KCNH1*-encoded human ethera`-go-go 1 (hEAG1) potassium channel was identified being responsible for higher cell proliferative rate in iPSC-derived MSCs using the Lian protocol [56, 58].

A new method to rapidly derive MSC-like cells from human iPSCs in one step using thin, fibrillar, type I collagen as matrix that mimics the structure of physiological collagen was reported for the effective differentiation of MSC from the human dermal fibroblast-derived HDFa-YK26 iPSCs [32]. Resilient colonies of homogenous spindle-shaped cells were obtained after 10 days of culturing iPSCs in alpha-MEM supplemented with 10% FBS, magnesium L-ascorbic acid phosphate, and dexamethasone that displayed the classical MSC characteristics [32]. After 2 passages, 82.9% of the cells were CD90-positive, indicating an efficient MSC generation. Prolonged passaging on collagen type I further increased this number to 96.9% of generated MSCs. The advantage for using collagen type I or in general appropriate biomaterial matrices here offers additional means to influence cell fate through physicochemical stimulation, as collagen type I was already known to activate an epithelial-to-mesenchymal transition (EMT) of epithelial cells [32, 59]. Another single-step method to direct mesengenic differentiation of human iPSCs was reported by Chen et al., who used a small molecule inhibitor, the transforming growth factor-*β* pathway inhibitor SB431542 [60]. iPSCs (MR90CL2 and ES4CL1) were cultured in the presence of this inhibitor for 10 days resulting in MSCs which exhibit typical MSC characteristics that conform to the criteria of the International Society for Cell Therapy (ISCT) for classification of MSCs. Mechanistically, this study revealed that SB431542 treatment triggered both intrinsic and autocrine mechanisms in iPSCs that collectively prime a subset of cells for a mesenchymal stromal cell fate by inducing EMT [60]. This modification in terms of fostering EMT in iPSC cultures resulted in higher MSC numbers of approximately 75–96% [60]. A simplified and reproducible method for inducing iPSC into MSC-like cells that further resulted in increased percentages of generated MSCs was then presented by Hynes et al. [61]. Herein, MSC-like cells were developed from iPSC lines arising from three different somatic tissues (gingiva, periodontal ligament, and lung) by a continuous culturing of respective cells in MSC culture media (alpha-MEM supplemented with 10% FCS, sodium pyruvate, l-ascorbate-2-phosphate, nonessential amino acids, and HEPES). After 2 weeks, the resulting heterogeneous cell types were passaged as a single-cell suspension and clones of arising cells were selected based on their typical morphology. Selected clones expressed key MSC-associated markers (CD73, CD90, CD105, CD146, and CD166) and lacked expression of pluripotent markers (TRA160, TRA181, and alkaline phosphatase) and hematopoietic markers (CD14, CD34, and CD45). *In vitro*, iPSC-MSC-like cells displayed the capacity to differentiate into osteoblasts, adipocytes, and chondrocytes [61]. By this method, the authors reported the generation of 95% pure MSC cultures.

Most of the protocols used fetal bovine serum as supplement which provides multiple growth factors with nonspecific signals to the cultures. In contrast, providing a chemically defined medium with known morphogens that fosters the MSC differentiation is supposed to increase the yield and homogeneity of the derived MSCs. In addition to the more specific and defined medium supplements, the use of xeno-free supplements (e.g., no animal products and no coculturing with mouse cells) would allow the generation of highly identical and clinically compliant MSC cultures from human iPSCs. Luzzani et al. used H9 human embryonic stem cells and iPSCs reprogrammed from human foreskin fibroblasts in combination with platelet lysate as a media supplement to produce pluripotent-derived MSCs (PD-MSC) within 3 to 4 weeks in a robust and consistent way [62]. The authors designed a two-stage protocol for the MSC differentiation from pluripotent stem cells. In the first step, mesodermal differentiation was induced by dissociating the pluripotent stem cell clusters and plating single-cell isolates on a reduced growth factor basement membrane matrix (Matrigel or Geltrex, a soluble and LDEV-free form of basement membrane extracted from murine Engelbreth-Holm-Swarm tumors) for 14 days in the presence of platelet lysate and supplement B27 [63]. After these 14 days, when the cells were transitioned to a mesenchymal state, the prolonged culturing was performed with plastic dishes in alpha MEM supplemented with platelet lysate (10%) for additional 7–14 days [62]. The resulting PD-MSCs were generated more efficiently as compared to cells differentiated in the presence of FBS as supplement (25 cells per pluripotent stem cells when platelet lysate was used versus 10 cells per pluripotent stem cells when FBS was used) and displayed all the MSC characteristics. Conclusively, the presented protocol used simple steps using therapy-grade platelet lysate as supplement and thus yielded significant amounts of MSCs in approximately 1 month [62]. Human serum in general as well as the derived platelet lysate (and thrombin-activated platelet releasate in plasma) turned out to be promising alternatives to FBS as a medium supplement for growing MSCs [64–66]. Although the concentrations of cytokines and growth factors in the respective supplements released by the platelets after lysing vary enormously, PDGF-AB/BB, TGF-*β*1, and bFGF turned out to be the essential factors (beside HGF and IGF-1) for the strong positive effect on the proliferation of MSCs [67, 68]. A potential but due to the strict protocols for blood testing minimal risk remains that the human material may contain virus or parasites [69].

In summary, robust protocols have been established to obtain patient-specific, therapeutically active MSCs from iPSCs in large amounts which will potentially open avenues towards novel, MSC-based therapies. Another straight forward strategy would be the ectopic overexpression of MSC-related genes or transcription factors in human iPSCs to generate MSCs. The *in vitro* generation of vascular wall-typical MSCs from iPSCs, based on a vascular wall MSC-specific gene code, was reported by our group [55, 70]. Herein, a lentiviral vector expressing a small set of recently identified human vascular wall MSC-specific HOX genes was used to directly program iPSCs into MSCs which displayed classical MSC characteristics, both *in vitro* and *in vivo* [55]. However, this forward programming approach remains limited to murine iPSCs, but it is very likely that our results will also hold true for human iPSCs as the activity of homeotic selector proteins is highly conserved throughout evolution.

## 5. Direct Conversion of MSCs from Somatic Cells

The main limitation for a possible therapeutic use of pluripotent stem cells and/or their derived MSCs is the medical risk to generate teratomas. Although already robust selection markers and refined experimental protocols have been established to guide human iPSCs reproducibly to MSCs, an additional negative selection against remaining pluripotent cells could be an additional option, to limit the risk of teratoma formation and foster clinical safety. As an alternative, somatic donor cells that have reached a particular differentiation stage could be used.

Meng et al. used CD34-positive cord blood and adult peripheral blood cells in combination with a single factor, namely, OCT4 to demonstrate a direct programming of patient-specific somatic cells into MSCs [71]. An episomal or lentiviral vector-mediated OCT4 expression followed by a subsequent culture of treated cells on fibronectin in commercially available MSC Medium Kit allowed the rapid and efficient programming of human CD34(+) cells directly into MSCs. The generated MSCs were multipotent, being able to differentiate into different types of MSC progenies both *in vitro* and *in vivo*, and were not tumorigenic [71, 72]. Conformingly, Lai et al. established an effective protocol to directly convert primary human dermal fibroblasts into multipotent, induced MSC-like cells (iMSCs) [73]. A cocktail containing six chemical inhibitors (SP600125 (JNK inhibitor), SB202190 (p38 inhibitor), Go6983 (protein kinase C inhibitor), Y-27632 (ROCK inhibitor), PD0325901 (ERK1/2 inhibitor), and CHIR99021 (GSK3*β* inhibitor)) with or without the addition of three growth factors (TGF-*β*1, bFGF, and leukemia inhibitory factor (LIF)) efficiently generated functional iMSCs from human primary dermal fibroblasts (primary neonatal foreskin fibroblasts (CRL2097) within 6 days (average rate of 38%)). The generated MSCs shared similar molecular signatures with bone marrow MSCs and fulfilled all of the MSC criteria defined by ISCT, including plastic adherence, marker expressions, and multipotency differentiation. *In vivo*, a markedly attenuation of endotoxin-induced acute lung injury, which was paralleled by a decrease of the amounts of proinflammatory cytokines, was reported [73]. Thus, an efficient conversion method that does not involve any processes that may lead to insertional mutagenesis, resulting in MSCs with lower safety concerns for disease treatments was reported [73].

Conclusively, chemical-induced conversion or direct programming of somatic cells into MSCs is possible and augurs strong clinical potential for respective MSCs but the protocols up to now are limited.

## 6. The Pros and Cons

Global gene and miRNA profiling of human iPSC- and embryonic stem cell-derived MSCs demonstrated a high degree of similarity between the derived MSCs, in particular as compared to bone marrow MSCs [45, 46, 49]. Bone marrow MSCs generally serve as the gold standard against which other MSC sources are compared [74]. However, there is a convincing evidence that MSCs from diverse tissue are different and display distinct differentiation tendencies, paracrine potential, and immune properties, but the benefit and mechanisms of these MSCs from various sources remain unexplored [75]. The significant heterogeneity in the differentiating potential of MSCs from different sources however may influence their clinical application [74, 76]. Adipose tissue-derived MSCs, for example were, shown to have a stronger inhibitory effect in the suppression of peripheral blood B, T, and NK cells than bone marrow and umbilical cord matrix-derived MSCs [77]. As another example, MSCs isolated from the placenta and adipose tissue were morphologically and immune phenotypically similar to MSCs obtained from the bone marrow, but MSCs derived from the placenta were proven to be a more optimal cellular source for the treatment of ischemic diseases [75]. A proteomic profiling of the three MSC types revealed that the highly upregulated proteins in placenta-derived MSCs, which were related to oxidative stress, peroxiredoxin activity, and apoptosis function, corresponded to the in vivo functional performance [75]. Previous reports have already demonstrated that bone marrow-derived MSCs were less effective after a therapeutically application as compared to other stem cell sources [77–82].

According to the general guidelines, MSCs from distinct tissue origins have a large number of similarities concerning their characteristics, but the isolated MSCs remain a heterogeneous cell population until a clonal expansion. Up to now, it is not clear, whether tissue-resident MSCs are the progenies of one ancestor cell lineage or the results of parallel cell developmental events [83, 84]. The tissue-specific properties of MSCs were related to the expression profiles of HOX genes that are master regulators of regional specification and organ development [46]. These HOX expression profiles can be used to distinguish functionally distinct populations of MSCs, as shown for bone marrow, umbilical cord blood, and blood vessel-derived MSCs [55, 70, 85]. However, the precise mechanisms that regulate lineage specification of the isolated heterogeneous MSCs have been largely unexplored [86, 87]. The transcriptome analyses of human MSCs revealed that expressed transcripts encode for a diverse repertoire of proteins that regulate angiogenesis, hematopoiesis, cell motility, neural activities, and immunity which finally lead to the conclusion that single cells were unlikely to possess all properties. [87–89]. The different functional attributes were relegated then to distinct subpopulations. Therefore, more effort is needed to develop clinical manufacturing protocols that reproducibly generate functionally equivalent MSC populations.

The tissue-specific homing and the activities of isolated and cultured MSCs prior to transfusion are supposed to be based on an underlying transcriptional code caused by epigenetic memory allowing them to home back to the tissue they originally were derived from [53]. In line with these findings, it was shown that vascular wall-derived MSCs were more potent than bone marrow-derived MSCs to protect lung blood vessels from the adverse late effects of radiotherapy, which supports the assumption that tissue-specific stem cells support mainly the tissue type from which they originate because of their tissue-specific action [90, 91]. Therefore, a central advantage would be the use of tissue-specific MSCs for the protection and curative treatment of the same and/or similar affected tissue that in turn would require protocols for the derivation of tissue-specific MSCs. The generation of tissue-specific MSCs from somatic cells and/or iPSCs reprogrammed from somatic cells could be achieved by transient, ectopic expression of cell type-specific transcription factors, miRNAs, or by using of epigenetic modifiers, as shown for other cell types, such as neurons or hepatocyte-like cells [92–95].

The age of MSCs may also have a major impact on their therapeutic outcome, as the differentiation potential of MSCs decreases with age [96]. Aged MSCs showed decreased proliferation rates, higher oxidative damage, and cell senescence [97]. This would argue against the derivation of MSCs from adult somatic cell types, but MSC derived from pluripotent stem cells may overcome the fact that adult MSCs have limited proliferation and differentiation capabilities. It was already suggested that every pluripotent cell may become a MSC “by default,” an event that occurs spontaneously when pluripotent stem cells are cultured under less stringent pluripotent conditions [62, 69]. However, the respective percentages of MSCs generated by these culture conditions were lower (approximately 5%) as compared to more specific culture conditions with mesoderm-specific growth factors. Epigenetic instabilities or phenotypic switches after prolonged culture might also occur because the identity of derived MSCs may not be well inherited in human iPSCs and embryonic stem cell-derived MSCs, and further studies are needed to specify the nature of derived MSCs and if all of the derived MSCs correspond to the similar (tissue-specific) MSC type.

Finally, the use of pluripotent stem cell-derived MSCs could be limited due to allogenic rejection or teratoma formation. In particular, the clinical use of the successful generated MSCs from human iPSCs and embryonic stem cells has been hampered by the tumorigenic potential elicited by undifferentiated iPSCs potentially remaining in the differentiated cell population, the lengthy and inefficient differentiation process, and genomic instability due to suboptimal culture conditions. Observed differences and efficiencies in MSC generation might be based on a somatic memory of the different cell types used for iPSC generation [98–100]. In line with this hypothesis, it was also shown that the cellular origin influences the lineage differentiation propensity of human iPSCs [41, 101]. A possible solution to these drawbacks could be to directly program an easily accessible patient-specific somatic cell types towards MSCs. Although a direct programming of somatic cells into MSCs is possible, robust protocols for this derivation are limited.

## 7. Conclusion

Beside the abundant origins but low frequencies of tissue-specific MSCs, the potentials to generate patient-individualized MSCs with comparable properties that bypass the immunogenicity and ethical issues in therapeutically relevant numbers are central advantages of using somatic cells and iPSCs as MSC source. Concerning the proliferative and regenerative potency of generated MSC, iPSC-derived MSC may be superior to somatic cell-derived MSC because MSCs differentiated from iPSCs might be closer to fetal MSCs, since pluripotent stem cells represent the early time point in development. In contrast, the tumorigenic potential of undifferentiated iPSCs in a population of iPSC-derived MSCs is a significant safety concern for iPSC-related clinical applications. This risk might be further reduced by the use of MSCs derived from integration-free iPSCs. An alternative quite promising method to gain MSCs *in vitro* is the direct conversion of somatic cells, for example, easily accessible fibroblasts or blood cells. However, respective studies and standardized protocols used to prepare large-scale MSC as well as useful tests to compare their potency are limited up to now. The significant molecular and functional differences in the properties of the generated MSCs according to their different origins influence the respective therapeutic potential. Therefore, the identification of the (tissue-specific) nature of *in vitro* generated MSCs from pluripotent stem cells needs further investigations as well as the establishment of protocols which would allow the generation of tissue-specific MSCs. Finally, the somatic memory of iPSCs should be carefully considered before clinical translation. Up to now, there are no reports on the direct comparison of MSCs generated by different approaches, which must be investigated in future studies together with the clinical safety of different MSC sources. Concerning the patient-derived autologous MSCs generated *in vitro*, the respective genetic background should be a benefit for (disease) modeling studies, but the same genetic or acquired abnormalities that predisposed a patient to a particular disease will be persisted in the respective MSCs which might result in dysfunctional MSCs with reduced therapeutically activities.
